# A novel missense variant in *CEACAM16* gene causes autosomal dominant nonsyndromic hearing loss

**DOI:** 10.1111/ahg.12463

**Published:** 2022-03-16

**Authors:** Dejun Zhang, Jie Wu, Yongyi Yuan, Xiaohong Li, Xue Gao, Mingyu Han, Song Gao, Shasha Huang, Pu Dai

**Affiliations:** ^1^ College of Otolaryngology Head and Neck Surgery Chinese PLA General Hospital Beijing China; ^2^ Department of Otolaryngology Head and Neck Surgery The Second Hospital of Jilin University Changchun China; ^3^ State Key Lab of Hearing Science, Ministry of Education National Clinical Research Center for Otolaryngologic Diseases Beijing China; ^4^ Beijing Key Lab of Hearing Impairment for Prevention and Treatment Beijing China; ^5^ Department of Otolaryngology, Head and Neck Surgery, National Children's Medical Center/Beijing Children's Hospital Capital Medical University Beijing PR China; ^6^ Department of Otolaryngology PLA Rocket Force Characteristic Medical Center Beijing China; ^7^ Department of Otolaryngology South‐East Hospital Affiliated to Xiamen University Zhangzhou China

**Keywords:** autosomal dominant nonsyndromic hearing loss, CEACAM16, DFNA4B, next‐generation sequencing, tectorial membrane

## Abstract

**Aim:**

Autosomal dominant non‐syndromic hearing loss is a common sensorineural disorder with extremely high genetic heterogeneity. CEA antigen‐related cell adhesion molecule 16(CEACAM16)is a secreted glycoprotein encoded by the CEACAM16 gene. Mutations in CEACAM16 lead to autosomal dominant non‐syndromic hearing loss in humans, due defects in the tectorial membrane of the inner ear. Here we reported a novel missense variant in CEACAM16 gene causes autosomal dominant non‐syndromic hearing loss.

**Material and methods:**

A four‐generation Chinese family affected by late‐onset and progressive hearing loss was enrolled in this study. The proband was analyzed by targeted next‐generation sequencing and bioinformatic analysis. And in vitro experiments were performed in overexpressed transfected HEK293T cells to investigate the pathogenesis of the mutant protein.

**Results:**

We identified a novel missense variant in the CEACAM16 gene c.763A>G; (p.Arg255Gly) as causing autosomal dominant non‐syndromic hearing loss in the Chinese family. Using Western blot analysis, ELISA, and immunofluorescence we found increased expression level of the secreted mutant CEACAM16 protein, both intracellularly and extracellularly, compared with wild type CEACAM16 protein.

**Conclusion:**

Our study showed that the p.Arg255Gly variant leads to increased secretion of mutant CEACAM16 protein, with potential deleterious effect to the function of the protein. Our findings expand the mutation spectrum of CEACAM16, and further the understanding CEACAM16 function and implications in disease.

## 1 INTRODUCTION

Hearing loss is the most common sensorineural disorder in humans. It is estimated that more than half of cases of hearing loss are attributable to hereditary causes. Autosomal dominant nonsyndromic hearing loss (ADNSHL) is a paradigm of genetic heterogeneity with more than 45 causative genes identified to date (http://hereditaryhearingloss.org/). These genes play an essential role in the morphology and development of hair cells, synaptic transmission of the auditory nerve, and other roles in the inner ear. For example, the transmembrane channel‐like gene1 (*TMC1*) is specifically required for hair cell mechanoelectrical transduction and is a functionally redundant stereocilia component (Kawashima et al., 2011; Pan et al., 2013) and eyes absent 4 (*EYA4*), a member of the vertebrate EYA gene family of transcriptional activators, is important for the development and maturation of the organ of Corti (Wayne et al., 2001). However, many genes causing deafness remain unidentified (Ding et al., 2020; Fu et al., 2021; Qian et al., 2020; Zhang et al., 2021; Zhu et al., 2018). It is therefore important to identify these genes and their functions in the inner ear.

Carcinoembryonic antigen‐related cell adhesion molecule 16 (*CEACAM16*, MIM 614591) is a member of the CEACAM family, which is known to play a role in tissue architecture and homeostasis, cell growth and differentiation, angiogenesis, and tumor suppression (Kuespert et al., 2006). *CEACAM16* maps to the DFNA4B locus on chromosome 19q13.32 and encodes a protein of 425 amino acids. Although the specific functions of the protein encoded by *CEACAM16* are still not clear, available evidence indicates that *CEACAM16* is crucial for hearing maintenance as a structural component of the tectorial membrane (Kammerer et al., 2012). *CAMCAM16* mutations can lead to late‐onset bilateral progressive sensorineural hearing loss that begin in the first or second decade (Wang et al.,2015). To date, three *CEACAM16* missense variants associated with ADNSHL have been reported (Hofrichter et al., 2015; Wang et al., 2015; Zheng et al., 2011). Additionally, Booth et al. (2018) and Dias et al. (2019) described loss of function variants in *CEACAM16* that can cause autosomal recessive nonsyndromic hearing loss (ARNSHL).

In this study, we identified a novel missense variant in the *CEACAM16* gene, c.763A>G; (p.Arg255Gly) in a Chinese family using targeted next‐generation sequencing approach. The phenotype was consistent with ADNSHL. Further functional experiments were carried out to evaluate the pathogenesis resulting from this mutation.

## 2 MATERIALS AND METHODS

### 2.1 Clinical evaluation

A four‐generation Han family M404 with ADNSHL from Xinjiang was recruited to participate in this study. The study was approved by the Chinese PLA General Hospital Research Ethics Committee. Written informed consent was obtained from the participants or the parents of minors. Comprehensive clinical information including the subjective degree of hearing loss, age of onset, medication, noise exposure, pathological changes in the ear, and other relevant clinical manifestations were obtained using a questionnaire. Four affected individuals (III‐6, III‐13, IV‐2, and IV‐3) were evaluated via otoscopy, physical examination, immittance testing, and pure tone audiometry according to standard protocols. The degree of hearing loss was assessed based on the 1997 WHO 1997 criteria. A computerized tomography (CT) scan of the temporal bone was also conducted in one proband (IV‐2).

### 2.2 Targeted sequencing and variation analysis

Genomic DNA of the proband (IV‐2) was extracted from peripheral blood using a blood DNA extraction kit following the manufacturer's instructions (TianGen, Beijing, China). A deafness panel containing 168 known deafness genes was used (Table S1). Capture and NGS of the coding exons of deafness genes and their flanking 100 bps were performed on an Illumina HiSeq 2000 by MyGenostics Corporation (Beijing, China). Sequencing reads were aligned to the human reference genome hg19 using the Burrows‐Wheeler Aligner. SAMtools, Picard, GATK, and ANNOVAR were used for genotype calling and annotation. ExomeDepth software was used to detect copy number variations. Variants were further annotated using public databases as follows: Exome Aggregation Consortium (ExAC) (Pawliczek et al., 2018); the 1000 Genomes Project; dbSNP (v.144) (Pawliczek et al., 2018); Genome Aggregation Database (gnomAD) (Gudmundsson et al., 2021); ClinVar (Pawliczek et al., 2018); the Human Gene Mutation Database (HGMD), and Deafness Variation Database (http:// deafness variation database. org/) (Azaiez et al., 2018). Pathogenicity of missense variants were predicted using the PolyPhen‐2, SIFT, LRT, MutationTaster, MutationAssessor, FATHMM, VEST3, and CADD programs. Multiple sequence alignment was performed according to a Homologene program.

### 2.3 Sanger sequencing

Genomic DNA was extracted from peripheral blood of family members. Sanger sequencing was performed to validate the filtered candidate variants and examine cosegregation of the genotype and phenotype. A total of 200 negative control samples from individuals with normal hearing were sequenced for candidate variants.

### 2.4 Plasmid DNA construction

The commercialized wild type plasmid DNA of *CEACAM16* (pCMV6‐*CEACAM16*−Flag) was purchased from a biotechnology company (RC224965;OriGene). The mutant type Arg255Gly of CEACAM16 was generated using the primers: 5′‐CTGTGGTGCGTGTCCgGGTCCTGCCC‐3′ and 5′‐CGGACACGCACCACAGGGTGAGGGAC‐3′. Then, the coding sequence was inserted into pCMV6‐Flag to construct the Arg255Gly mutant type plasmid. All constructs were verified by Sanger sequencing.

### 2.5 Cell transfection and immunofluorescence

HEK293T cells were transfected with pCMV6‐*CEACAM16*‐Flag and pCMV6‐Arg255Gly‐Flag plasmid using Lipofectamine 3000 reagent for overexpression of CEACAM16 and Arg255Gly, respectively. Transfection with empty pCMV6 served as negative control. Transfected HEK293T cells were fixed in 4% paraformaldehyde, permeabilized in 0.2% PBST, and blocked in 10% goat serum. Cells were incubated with the primary antibody anti‐CEACAM16 (45854‐1;SAB), and then with the secondary antibody anti‐α‐SMA (ab5694; Abcam. Goat anti‐rabbit IgG‐Alexa Fluor 488 (ab150077;Abcam) and anti‐Mouse IgG‐HRP (Ab150116;Abcam) were for detection of the primary antibody. Cells were examined with a laser scanning confocal microscope.

### 2.6 Western blot analysis

The culture medium from CEACAM16‐transfected cells was collected and centrifuged at 12000 × rpm for 20 min, at 4°C. Protein extraction was performed and protein samples were transferred onto polyvinylidene difluoride membranes. Due to the low protein content in the culture medium, we used Milipore ultrafiltration tubes to allow concentration for western blot analysis. Membranes were blocked in Tris‐buffered saline supplemented with 5% nonfat milk for 2 h, at room temperature. Incubation with primary antibodies was performed at 4°C, for 18 h. The antibodies used were as follows: anti‐GAPDH (1:5000; 100494‐1‐AP; Proeintech) and anti‐CEACAM16 (1:1000; 45854‐1; SAB). Cells were further incubated with goat anti‐rabbit IgG (1:5000; ZB2301, ZSGB‐Bio, Beijing, China), and goat anti‐mouse IgG (1:5000; ZB2305, ZSGB‐Bio, Beijing, China) HRP‐ conjugated antibodies for 2 h, at 24°C. Signal detection was performed using the ECL and western blotting detection system (GE Healthcare) according to the manufacturer's instructions. Finally, immunoblots were analyzed using the Image Lab (ImageJ v 1.8.0 version) and Graphpad Prism 7.0) software. GAPDH was used as an internal reference and the relative gray value of each index was calculated.

### 2.7 Validation of enzyme linked immunosorbent assay

Levels of wild type and mutant Arg255Gly CEACAM16 proteins extracted from culture medium were quantified using a human CEACAM16 enzyme linked immunosorbent assay (ELISA) Kit (JL47689; Jianglai‐Bio), following the manufacturer's instructions. Experiments were performed in triplicate. Briefly, 50 μl protein samples at different concentrations were added to different wells, and blocked with 100 μl of HRP labeled antibody for 1 h, at 37°C. The solution was removed, and samples were washed five times with 1× Wash solution. Note that when 50 μl of substrates A and B were added to each well, incubation was done at 37°C for 15 min. Finally, 50 μl stop solution was added to each well and absorbance was measured at λ450 nm using an ELISA reader.

### 2.8 RNA extraction and quantitative real‐time PCR

Wild type and mutant *CEACAM16*‐transfected cells were washed with PBS and retrieved for RNA extraction. Total RNA was isolated using Trizol Reagent according to the manufacturer's instructions. Real‐time PCR assay was performed using SYBR green dye on Step One sequence detection system (Applied Biosystems, Waltham, MA, USA). The relative abundance of CEACAM16 mRNA was calculated using the 2−ΔΔCT formula, and with GAPDH as internal control. Primers were as follows: CEACAM16 forward primer 5′‐GACCACCTCAACATCAGCAGCAT‐3′, CEACAM16 reverse primer 5′‐ GGTCTTGGTGTTCTTCGCAATACATG‐3′; GAPDH forward primer 5′‐TCAAGAAGGTGGTGAAGCAGG‐3′, GAPDH reverse primer 5′‐GCGTCAAAGGTGGAGGAGTG‐3. All experiments were performed in triplicate.

### 2.9 Statistical analysis

Differences among groups were assessed by one‐way ANONA and Student's *T*‐test. Statistical significance was defined as*p < 0.05, ** *p* < 0.01 and ***p < 0.0001.

## 3 RESULTS

### 3.1 Clinical findings

**FIGURE 1:**
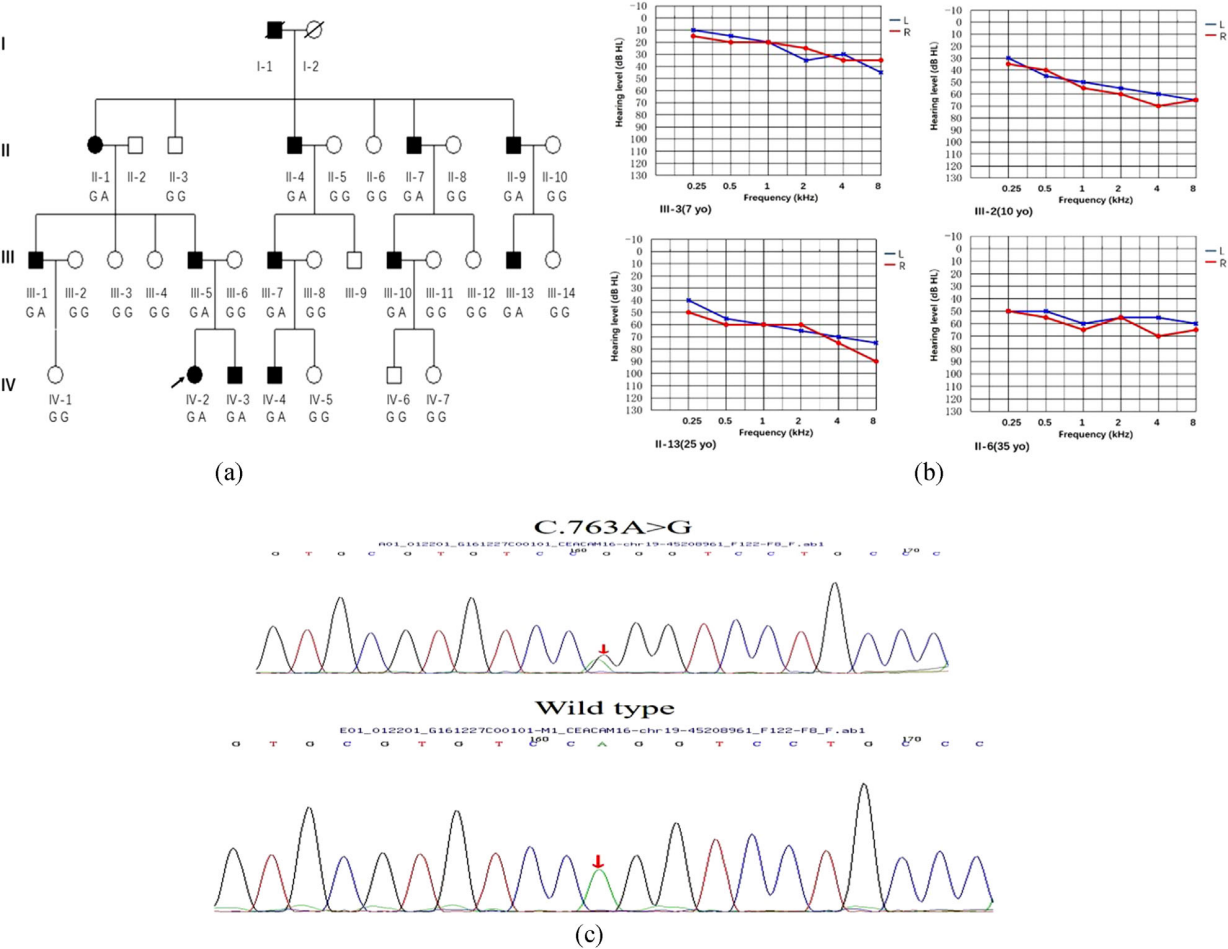
(a)Pedigree of Chinese family M404. The pedigree shows an autosomal‐dominant inheritance pattern. Affected subjects are denoted in black. The proband is indicated by an arrow. The deceased are differentiated by a slash. (b) Audiograms of four affected subjects showed bilateral sensorineural hearing loss (IV:3, IV:2, III:13 and III:6). Hearing levels of the right and the left ear are marked using red and blue lines, respectively. (c) Deafness gene panel identified a *CEACAM16* c.763A>G(p.Arg255Gly) variant in the proband and this variant was validated by Sanger sequencing in all the affected individuals and was absent from 200 normal hearing controls

The M404 family evaluated in this study included 12 affected individuals and 19 unaffected members (Figure 1a). The proband (IV‐2) was a 10‐year‐old girl who had bilateral hearing loss detected 2 years ago. Audiometry showed a down‐sloping configuration (Figure 1b). Interestingly, her younger brother (IV‐3), a 7‐year‐old boy, had no noticeable hearing loss, but the audiogram revealed high‐frequency hearing loss. Both the father and uncle (III:6 and III:13) of the proband presented with bilateral moderately severe sensorineural hearing loss, which had started around the age of 10 and got progressively worse with age. The other affected family members did not undergo a hearing test, but, according to information provided by other family members, it could be inferred that they also experienced bilateral progressive hearing loss, which varied from mild to profound degree, with onset during the first decade of life.

A high‐resolution CT scan on the proband revealed no alterations. Physical examination of the family members revealed no signs of systemic illness or dysmorphic features. None of the affected individuals reported complains consistent with tinnitus or vestibular dysfunction.

### 3.2 Molecular analysis

Using the 168 deafness gene panel, we sequenced all coding exons and approximately100 bp of flanking intronic sequence in the proband. The average depth of coverage reached 410.24×, with 98.16% of sequences having coverage greater than 4×, 97.62% greater than 10×, and 96.99% greater than 20×. Variants were identified (Table S2), and examined for consistency with the autosomal dominant inheritance pattern. Only dominant genes and variants were selected as candidates. Candidates in *CEACAM16*, *COL11A1*, *COL9A2, DIAPH1, TCOF1* genes were filtered using different databases for a minor allele frequency lower than 0.005, and their pathogenicity was predicted using several prediction programs. The remaining gene variants in *CEACAM16*, *COL11A1*, *COL9A2*, *DIAPH1*, *TCOF1* were tested for segregation with the hearing loss phenotype in the extended family. Segregation analysis revealed that only the heterozygous A‐to‐G transition at position 763 in exon 5 of *CEACAM16* (NM_001039213: c.763A>G; (p.Arg255Gly) cosegregated with the hearing loss phenotype (Figure 1a). The c.763A>G variant was absent in the ExAC, 1000 Genomes project, dbSNP, ClinVar, and HGMD, and was also absent from the 200 ethnicity‐matched normal hearing controls. However, this variant was described as likely benign in Deafness Variation Database.

### 3.3 In silico variant analysis

**FIGURE 2:**
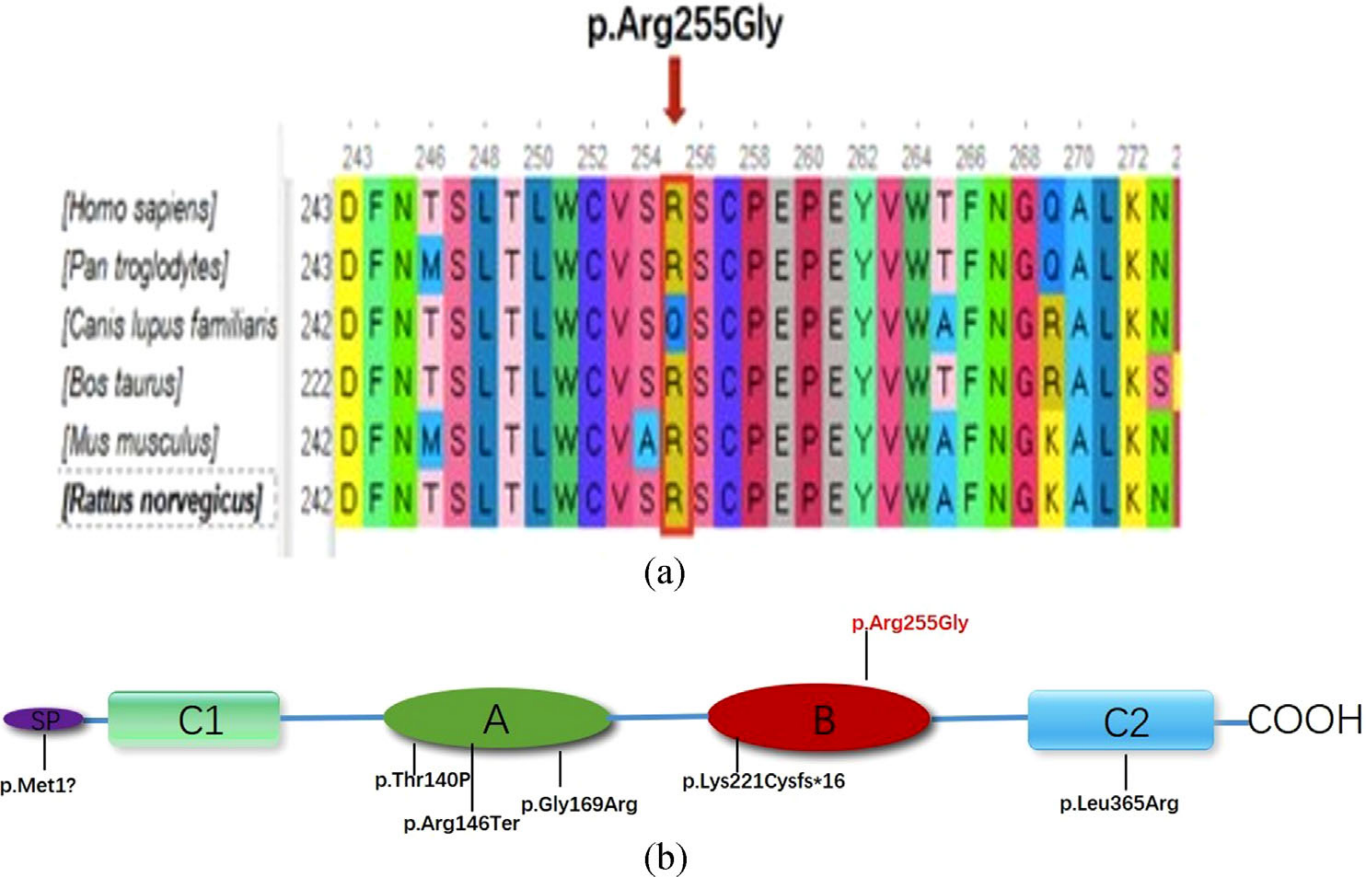
(a) Multiple sequence alignment showing that Arg255 in CEACAM16 is conserved in human, chimpanzee, cow, mouse and rat, but not in dog. (b) Schematic structure of CEACAM16: A, B, C1, C2 and SP represent IgC‐like domain A, IgC‐like domain B, IgV‐like domain N1, IgV‐like domain N2 and signal peptide, respectively. The location of five reported CEACAM16 variants (bottom) and p.Arg255Gly(top) identified in this study is marked on the diagram

Multiple sequence alignment was performed using ClustalW2 using the default settings, revealing conservation of the mutant amino acid loci in human, chimpanzee, cow, mouse, and rat, but not in dog (Figure 2a).

The c.763A>G variant was analyzed using eight deleteriousness prediction programs and was found to be benign, tolerable, unknown, polymorphism, low functional impact, tolerable, damaging, and damaging (Table 1).

**TABLE 1:** Summary of pathogenicity prediction and database analysis in the known mutations of CEACAM16

Prediction programs	c.763A>G	c.418A>C	c.505G>A	c.1094T>G	c.37G>T	c.662–1G>C
and databases	(This study)	(Zheng et al., 2011)	(Wang et al., 2015)	(Hofrichter et al., 2015)	(Booth et al., 2018)	(Dias et al., 2019)
PolyPhen‐2	Benign	Probably damaging	Probably damaging	Probably damaging	Benign	_
SIFT	Tolerable	Tolerable	Damaging	Damaging	Damaging	_
LRT	Unknown	Unknow	Unknown	Unknown	Unknown	_
MutationTaster	Polymorphism	Polymorphism	Polymorphism	Disease causing	Polymorphism	Disease causing
MutationAssessor	Low	Medium	Neutral	Low	Neutral	_
FATHMM	Tolerable	Tolerable	Tolerable	Tolerable	Tolerable	_
VEST3	Damaging	Damaging	Tolerable	Damaging	Tolerable	_
CADD	Damaging	Damaging	Tolerable	Damaging	Damaging	Damaging
GERP	Nonconserved	Conserved	Nonconserved	Conserved	Conserved	Conserved
phyloP	Nonconserved	Conserved	Nonconserved	Conserved	Conserved	Conserved
ExAC(Pawliczek et al., 2018)	_	_	_	_	_	_
1000 Genomes Project	_	_	_	_	_	_
dbSNP(Pawliczek et al., 2018)	_	_	_	_	_	_
gnomAD (Gudmundsson et al., 2021) 3.23e‐05	_	_	_	_	_	
ClinVar(Pawliczek et al., 2018)	_	Pathogenic	Pathogenic	_	_	_
HGMD	_	Pathogenic	Pathogenic	_	_	_
Deafness Variation Database						
(Azaiez et al., 2018)	likely benign	Pathogenic	Pathogenic	_	_	_

*Note*: Pathogenicity prediction programs include: PolyPhen‐2,SIFT, LRT, MutationTaster, MutationAssessor,FATHMM, VEST3, and CADD; Allele frequency in population databases: ExAC, 1000 Genomes Project, dbSNP, and gnomAD;Disease‐specific databases: ClinVar, HGMD, and Deafness Variation Database; −:data not present.

Variant classification was completed according to the Hearing Loss‐specific American College of Medical Genetics and Genomics (ACMG) guidelines (Patel et al., 2021). The c.763A>G variant cosegregated with hearing loss in the family (PP1_Strong) and was absent from multiple population databases and the 200 normal hearing control cohort (PM2). In vitro functional experiments showed that this variant impaired protein function (PS3_Surpporting). Therefore, *CEACAM16* p.Arg255Gly fulfills PS3_Surpporting, PM2, PP1_Strong evidence, and could be classified as a “Likely Pathogenic” according to the ACMG guidelines.

### 3.4 Functional analysis of p.Arg255Gly

**FIGURE 3:**
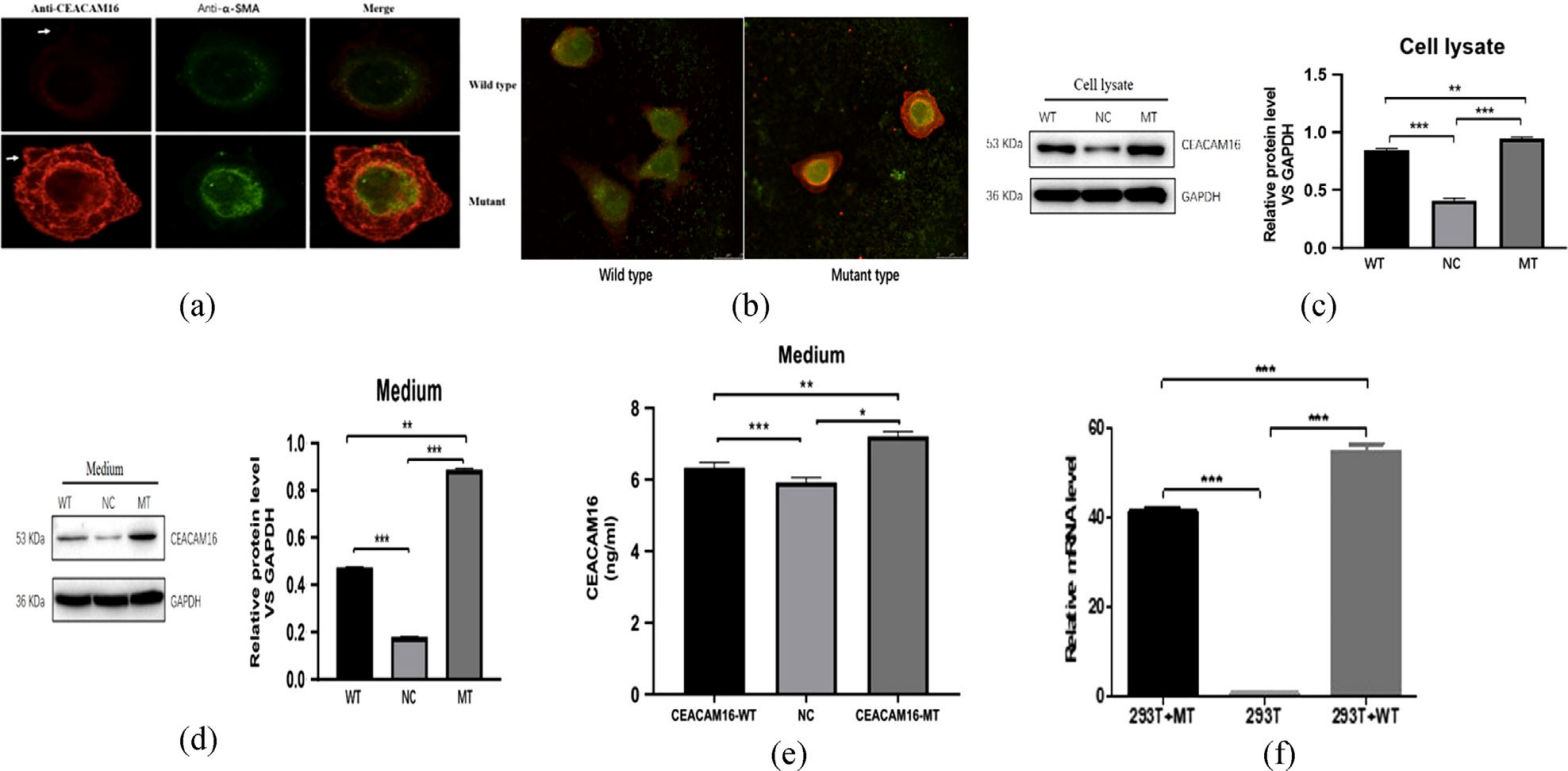
(a) Immunofluorescence analysis of HEK293T cells expressing WT and Arg255Gly mutant CEACAM16 (red). Counterstained was performed with α−SMA(green, scale bar, 10 μm). White arrows show secreted proteins. (b) Immufluorescence image of multiple HEK 293T cells expressing WT and mutant CEACEAM16. (c) Western blot of the expression of CEACAM16 in cell lysate, and the relative protein level among WT, NC, and MT groups. (d) Western blot of the expression of CEACAM16 in culture medium, and the relative protein level among WT, NC, and MT groups. (e) The quantitative protein levels of CEACAM16 in culture medium determined by ELISA. (f) CEACAM16 mRNA expression in cell lysate was examined by quantitative real‐time PCR, the expression levels relative to those of GAPDH were normalized. Values are expressed as the mean ± standard deviation (*n* = 3). WT: Wild Type, cells transfected with wild type of CEACAM16 plasmid; MT: Mutant Type, cells transfected with mutant Arg255Gly CEACAM16 plasmid; NC: Negative Control, cells transfected with empty plasmid; ns: no statistical significance; *p < 0.05, ** *p* < 0.01 and ***p < 0.0001

To investigate whether p.Arg255Gly affects CEACAM16 protein function, wild‐type (WT) and mutant pCMV6‐Arg255Gly‐Flag plasmids were transiently transfected into HEK293T cells. Expression and localization of WT and mutant proteins was done using immunofluorescence. As shown in Figure 3a and B, WT and mutant CEACAM16 proteins were mainly distributed in the cell cytoplasm, without regional aggregation, with no apparent differences in their subcellular localization. However, in comparing their expression levels, the level intracellular and extracellular mutant proteins seems increased compared with wild‐type proteins.

Next, in order to evaluate the changes of mutant CEACAM16 proteins, protein collected from the cell lysate and culture medium was analyzed using western blot. Similar to the WT protein, an immuno‐reactive band of molecular weight 53 kDa corresponding to the mutant CEACAM16 protein was detected in both samples from the cell lysate and culture medium. The expression level of the mutant CEACAM16 protein was higher compared with the WT, consistent with our previous observation using immunofluorescence methods (Figure 3c,d). In addition, ELISA assays were used to measure the CEACAM16 protein amount in the culture medium. The amount of mutant CEACAM16 protein was significantly higher than that of WT protein (*p* < 0.01) (Figure 3e).

Quantitative real‐time PCR (RT‐qPCR) was performed to detect the relative expression level of *CEACAM16* mRNA. As shown in Figure 3e, the mRNA expression level of *CEACAM16* p.Arg255Gly group was significantly lower than that of the WT group (*p* < 0.001), suggesting that the Arg255Gly mutation does affect the function of CEACAM16 as a secreted glycoprotein.

## 4 DISCUSSION

The tectorial membrane is essential for normal hearing due to its exclusive physical properties and its role in modulating the flow of fluid in the subtectorial space (Nowotny & Gummer, 2011), amplifying the basilar membrane motion (Booth et al., 2019; Zwislocki & Kletsky, 1979), and shaping the cochlear tuning (Teudt & Richter, 2014). The tectorial membrane is composed of collagen and noncollagenous proteins including alpha‐tectorin (TECTA), beta‐tectorin (TECTB), otogelin (OTOG), otogelin‐like (OTOGL), and CEACAM16. Research in both humans and animal models have shown that pathogenic variations in genes encoding for these proteins can alter the properties of the tectorial membrane and eventually lead to hearing loss (Cheatham et al., 2014; Ghaffari et al., 2013; Legan et al., 2014; Schraders et al., 2012; Yariz et al., 2012).

CEACAM16 protein is one of the proteins of the tectorial membrane which is thought to be essential for its function. As mentioned earlier, CEACAM16 is a secreted glycoprotein encoded by *CEACAM16* gene and belongs to the CEACAM immunoglobulin superfamily (Zheng et al., 2011). Unlike other members of the family, CEACAM16 is well conserved in mammals and is selectively expressed in the inner ear (Kammerer et al., 2012). The structure of the CEACAM16 protein (Figure 2b) contains two Ig constant‐like domains (domain A and domain B) and two Ig variable‐like domains (domain N1 and domain N2) with the N‐ and the C‐terminus separately, and lacks both a C‐terminal transmembrane domain and a glycosylphosphatidylinositol anchor (Zebhauser et al., 2005). Its unique structure allows crosslink with TECTA and TECTB which involves the formation of the striated‐sheet matrix, a laminated component that associates with the frequency‐dependent mechanical properties of the tectorial membrane (Goodyear & Richardson, 2018; Jones et al., 2015). One study observed that the striated‐sheet matrix was absent from the tectorial in the Ceacam16^βgal/βgal^ mice (Cheatham et al., 2014). Data from mice lacking Ceacam16 found high and low frequency hearing loss in young mice (Kammerer et al., 2012), and in humans, a similar phenotype was reported in families harboring variants in the *CEACAM16* gene (Booth et al., 2018; Hofrichter et al., 2015; Wang et al., 2015; Zheng et al., 2011).

In our research, we identified a missense variant in *CEACAM16* (p.Arg255Gly) which caused ADNSHL in a Chinese family. All affected individuals showed bilateral progressive sensorineural hearing loss, a very similar phenotype to the one reported in an American family (1070) and another Chinese family (SY‐026) (Wang et al., 2015; Zheng et al., 2011). However, the earliest onset age of hearing loss in the family reported in our study was 7 years old (IV‐3), substantially earlier than that reported in the other families (the youngest was 10 years old) (Booth et al., 2018; Hofrichter et al., 2015; Wang et al., 2015; Zheng et al., 2011). These findings suggests that the onset of hearing loss caused by the *CEACAM16* variants can occur within the first decade of life in humans. These findings appear consistent with those seen in the Ceacam16 null mouse, which also displayed hearing loss at a young age (∼4 weeks old) (Kammerer et al., 2012). Based on our bioinformatic analysis, the p.Arg255Gly variant cosegregated with the phenotype of DFNA4B in the family and was absent from multiple population databases (Table 1) and the 200 normal hearing controls cohort. The arginine residue at 255 in CEACAM16 was not found to be highly conserved across species (lack of conservation in dogs) (Figure 2A). The p.Arg255Gly variant is located within the Ig constant‐like domain B (Figure 2B), which is crucial for stabilizing the Ig‐like conformation and increasing the affinity of the homophilic binding (Athanasia & Andreas, 2014; Bonsor et al., 2015). Therefore, we speculate that this novel missense variant might damage the structure and function of CEACAM16.

Transfection of the mutant protein (p.Arg255Gly) in HEK293T cells, visualized using immunofluorescence, suggest similar intracellular and extracellular protein expression between mutant and wild type proteins. These results are in agreement with our findings using Western blot, and suggest that the p.Arg255Gly variant does not interfere with the normal subcellular localization of CEACAM16. Our findings are also consistent with those reported by Wang et al. (2015) and Zheng et al. (2011). Furthermore, we performed quantitative analysis of the mutant proteins extracted from the cell lysate and culture medium by Western blot and ELISA, respectively. Interestingly, in both cases the amount of mutant protein was higher than that of WT, indicating that the new variant in *CEACAM16* increases the secretion of the mutant CEACAM16 protein, and suggesting it is a gain‐of‐function mutation. Subsequently, in order to investigate whether the variant p.Arg255Gly affected protein expression at the transcription level, we used PCR to detect the relative expression l *CEACAM16* mRNA level in HEK293T cells. In contrast to the Western blot results, PCR results indicated that mRNA expression levels of mutant *CEACAM16* were significantly lower than those of the WT group. There may be several reasons for this inconsistency. First, the difference in plasmid transfection efficiency of transiently transfected HEK293T cells may have masked the true mRNA levels of the mutant gene. Second, according to the research of Perl et al. (2017), protein levels are more conserved than mRNA levels in all datasets, and changes in transcription are associated with translational changes that exert opposite effects on the protein level. Third, some other factors may influence the correlation between mRNA and protein expression, such as mRNA degradation rate, variation of mRNA secondary structure, among others (Guo et al., 2008). To date, the specific function of the protein encoded by *CEACAM16*, as well as the pathogenetic mechanisms underlying hearing impairment of patients harboring variants in this gene, remain unclear. In previous studies, a missense variant (p.Thr140Pro) in *CEACAM16* was identified in an ADNSHL pedigree and was predicted to disrupt a glycosylation site, interfering with protein stability (Zheng et al., 2011). In another family with hearing loss, the p.Gly169Arg variant at Ig‐C like domain A was assumed to change the spatial structure of CEACAM16 and decrease stability and the polymerizing ability of the protein (Wang et al., 2015). However, these studies lack in vivo evidence to help validate their assumptions. To the best of our knowledge, CEACAM16 is a secreted glycoprotein with a signal peptide at the N terminus; guided by the signal peptide, the newly synthesized glycoprotein is translocated to the endoplasmic reticulum and then secreted (Blobel & Dobberstein, 1975). According to Kammerer et al. (2012), CEACAM16 is located in Deiters and interdental cells of the organ of Corti in the inner ear of mice, and released via the Deiters cell's projections, after which is incorporated into the matrix of the tectorial membrane. In the tectorial membrane, CEACAM16 interacts with TECTA and TECTB via its two Ig‐V like domains where it forms the dark and light zone of the striated‐sheet matrix (Goodyear & Richardson, 2018). The p.Arg255Gly mutation we report occurs within the Ig‐C like domain B of CEACAM16. This Ig‐C like domain contains conserved cysteine residues that stabilize the Ig‐like conformation by forming a disulphide bridge (Bork et al., 1994; Williams & Barclay, 1988), and plays a key role in CEACAM16 dimer formation (Kammerer et al., 2012). By comparing the properties of arginine and glycine, we know that the mutant residue is smaller and more hydrophobic than the wild‐type residue, and is neutral in contrast to the wild‐type residue, which is negatively charged. This mutation introduces an amino acid with distinct properties, which can disturb the Ig‐C like domain B and decrease protein stability (Cheng et al., 2006; Venselaar et al., 2010). Therefore, we speculate that the variant p.Arg255Gly might cause structural changes in the CEACAM16 protein, altering its intracellular synthesis and transport pathways, and leading to increased secretion. At the same time, due to the decreased stability of the extracellular protein, the mutation might interfere with CEACAM16 dimerization and affect the interaction between CEACAM16 and other proteins. This alteration might also compromise the physical properties of the tectorial membrane and eventually lead to hearing loss. This study also has some limitations. We used CEACAM16 antibody commercial kits and were unable to detect oligomers using Western blot. Further vivo experiments are necessary to investigate the pathophysiological significance of CEACAM16.

In summary, we implicate a novel variant in *CEACAM16* as the genetic cause of progressive hearing loss in a large Chinese pedigree. We suggest that this variant leads to increased secretion of mutant CEACAM16 protein in vitro, uncovering a putative novel mechanism underlying DFNA4B‐related hearing loss.

## CONFLICT OF INTEREST

The authors declare no conflict of interest.

## AUTHOR CONTRIBUTIONS

Dejun Zhang, Shasha Huang, and Pu Dai carried out the studies, participated in collecting data, and drafted the manuscript. Jie Wu, Yongyi Yuan, and Xiaohong Li performed the statistical analysis and participated in its design. Xue Gao, Mingyu Han, and Song Gao helped to draft the manuscript and performed the data analysis. All authors read and approved the final manuscript.

## Supporting information

Supplemental Table 1 168 deafness genes

Supplemental table 2 Variants identified in the 168 deafness gene panel

## Data Availability

The data used and/or analyzed during the current study are available from the corresponding author on reasonable request.
